# CHALLENGES OF CONDUCTING COMMUNITY-ENGAGED RESEARCH AMONG JUSTICE-INVOLVED MID- AND LATE-LIFE ADULTS

**DOI:** 10.1093/geroni/igad104.0277

**Published:** 2023-12-21

**Authors:** Rodlescia Sneed

**Affiliations:** Wayne State University, Detroit, Michigan, United States

## Abstract

Formerly incarcerated mid and late-life adults exhibit high rates of chronic disease and disability; however, there is little attention to chronic physical health within programs designed to provide community reentry support. Healthy eating and physical activity are known to reduce chronic disease risk; yet existing physical activity and nutrition programs fail to address the needs of formerly incarcerated mid and late-life adults, who often experience housing insecurity, unemployment, low literacy, and limited access to healthy food. Through a partnership with a community-based reentry program, we designed a health and wellness program tailored to the needs of formerly incarcerated mid and late-adults that included group fitness instructor training, weekly nutrition/fitness classes, and peer coaching. Our team experienced numerous challenges in implementing this program, including low willingness of local community agencies to engage with justice-involved mid and late-life adults, competition with other reentry program activities, individual-level social factors that inhibited participation, and parole/probation restrictions that negatively impacted program attendance. In our session, we will describe how we engaged with a diverse range of partners to address these challenges to ensure program success. There are a number of barriers associated with conducting community-engaged research among formerly incarcerated mid and late-life adults. Our work to date will inform future engagement with this population.

